# A cross-sectional assessment of cervical cancer vaccination and screening among women of reproductive age living with HIV in North Central States, Nigeria

**DOI:** 10.11604/pamj.2025.52.175.49492

**Published:** 2025-12-19

**Authors:** Oluyemi Peter Atibioke, Beatrice Tomisin Oyasope, Oluwaseun Ayoola Ojomo, Bayo Waidi Jima, Kehinde Akewusola, Janet Ogunkayode, Fatimah Adepoju-Olajuwon, Bilqis Wuraola Alatishe-Muhammad, Nike Kehinde, Idris Saliu, Andrew Etsetowaghan, Ayodotun Olutola

**Affiliations:** 1 Programs Department, Association for Reproductive and Family Health, Ilorin, Nigeria,; 2 School of Medicine, University of Global Health Equity, Kigali, Rwanda,; 3 Severe Typhoid in Africa (SETA Project), University College Hospital, Ibadan, Nigeria,; 4 Department of Epidemiology and Community Health, Kwara State Ministry of Health, Ilorin, Nigeria,; 5 Clinical Services Department, Centre for Clinical Care and Clinical Research, Ilorin, Kwara State, Nigeria

**Keywords:** Cervical cancer, vaccine, screening, women living with HIV, knowledge, Nigeria

## Abstract

**Introduction:**

cervical cancer remains a major public health issue in Nigeria, particularly among women of reproductive age. This study investigated knowledge, risk perception, practices, and barriers to cervical cancer vaccination and screening among women living with HIV in North Central Nigeria. Understanding these factors is crucial for developing effective public health interventions.

**Methods:**

a cross-sectional study design was employed, involving 991 women of reproductive age randomly selected through a systematic sampling technique among those attending various ART clinics in Kwara and Niger States. Facilities were purposively selected among ART clinics across the three senatorial districts of each State. Data were collected using a 77-item semi-structured questionnaire and analysed using descriptive statistics, Chi-square, and Fisher's exact at α0.05.

**Results:**

despite the relatively high educational level among participants, 61.5% had never been screened for cervical cancer, and 96.3% had never been vaccinated. Cervical cancer awareness was high (76.2%), but comprehensive knowledge was low (34.6%). Key barriers included fear of pain, fear of positive results, logistic challenges, and cultural factors. Higher educational attainment and urban residency were associated with better knowledge and practices.

**Conclusion:**

there are significant gaps between awareness and actual practice of cervical cancer preventive measures among women living with HIV in North Central, Nigeria. Also, there exist various barriers that need to be addressed through various strategies highlighted in the study. These interventions are essential to increasing screening and vaccination rates and ultimately reducing the burden of cervical cancer among women of reproductive age in North Central Nigeria.

## Introduction

Cervical cancer is the fourth most common cancer in women globally. In 2018, an estimated 570,000 women were diagnosed with cervical cancer worldwide, and about 311,000 women died from the disease [1]. Cervical cancer, predominantly caused by high-risk human papillomavirus (HPV) infection, is a significant global health issue, particularly in developing countries, where prevention and early detection are essential to reducing its burden [2]. Cervical cancer is one of three cancers that define acquired immunodeficiency syndrome (AIDS), and cervical cancer diagnoses are more than three times higher among women living with HIV [3,4]. A major risk factor for developing HPV-caused precancerous lesions and progressing to aggressive cervical carcinoma is co-infection with HIV [1,3,5]. HPV infection that persists is linked to advanced HIV infection, which is characterised by a compromised immune system because of a decrease in cluster of differentiation 4 (CD4) T-helper cells, an indication of AIDS [6]. In Nigeria, the adult HIV prevalence is 1.3%, and higher among women between the ages of 15 and 49 at 1.6% [7]. Factors such as high HIV prevalence, limited access to preventive services, and low CD4 counts are exacerbated in low-resource settings due to poor healthcare infrastructure and low awareness. These factors explain why cervical cancer occurs in HIV-positive people living in low-resource settings. Cervical cancer is often deemed a “preventable” and “treatable” disease due to the availability of effective interventions, including HPV vaccination, routine Pap smear screening, and timely treatment of precancerous lesions and invasive cancers [8]. When implemented optimally, these strategies can significantly reduce cervical cancer incidence and mortality rates [9]. However, limited awareness of HPV and cervical cancer, low perceived risk, financial constraints, cultural beliefs, and inadequate healthcare access continue to hinder the utilisation of preventive services [1,10].

Despite ongoing efforts to promote cervical cancer prevention and early detection among women living with HIV (WLHIV). This demographic group continues to face a substantial burden of cervical cancer due to gaps in knowledge, inconsistent risk perceptions, and systemic barriers [11]. These challenges hinder the effectiveness of public health interventions and call for a comprehensive study that examines the multifaceted aspects of cervical cancer prevention and treatment among women of reproductive age in this region. However, there is a need for more in-depth investigations into the dynamics of these disparities and the factors contributing to them. Furthermore, the interplay between knowledge, risk perception, and actual healthcare utilisation in the context of cervical cancer remains underexplored. This study, therefore, sought to investigate the complex interplay of knowledge, perception, and practice regarding cervical cancer prevention among women of reproductive age living with HIV in North Central Nigeria. Furthermore, the study aimed to elucidate the specific socio-demographic, cultural, and logistical barriers that hinder the adoption of these life-saving interventions. By providing a nuanced understanding of these factors, this research endeavours to inform targeted interventions and policies that enhance cervical cancer prevention and control efforts among WLHIV in the North Central States.

## Methods

**Study design and setting:** a descriptive cross-sectional design was adopted for this study to investigate knowledge, risk perception, practice, and barriers to cervical cancer screening and vaccination among women of reproductive age in Kwara and Niger States, North Central Nigeria.

**Study population:** the study population consisted of women currently living with HIV who were of reproductive age (15-49 years). According to the electronic medical record (EMR) 'Radet' file as of September 2023, the total number of clients on treatment in the supported health facilities was 41.387. From this, the population of women was 28.143, of whom 23.077 were identified as women of reproductive age.

**Sample size determination:** the minimum sample required was obtained using the Kish-Leslie formula [12] for estimating sample size for a single population. The estimated value is obtained as shown below:


$$n=\frac{Z^{2}pq}{d^{2}}$$

Where: n= sample size; Z= the standard score (critical value) corresponding to a 95% confidence interval, which is equal to 1.96; d= the proportion of random sampling error between the sample and the population, which is chosen to be 5%; p= prevalence (50%; was used as a proxy), 1.962 x 0.34 x (1-0.34) /0.052 =384.16. Calculating for a 10% non-response rate 384.16 / (1-0.10) =427. The sample size of 427 was the minimum sample size applied for each of the two States.

**Inclusion and exclusion criteria:** the study included women aged 15-49 living with HIV/AIDS, accessing treatment in USAID-supported facilities in Kwara and Niger States. Excluded were women under 15 or over 49, men, children, those not living with HIV/AIDS, and those who did not consent or had conditions impacting their ability to respond effectively.

**Sampling technique:** a multistage sampling technique was adopted, and two States were selected purposively. Each State was divided into clusters by senatorial districts to select LGAs. Health facilities were chosen purposively, and respondents were selected systematically from each facility. The purposive sampling of the two States and the health facilities was necessitated by the operational scope of the implementing partner (IP). The study was conducted with partner organisations that implement the USAID-funded HIV project, and this project was active only within selected healthcare facilities providing ART services to people living with HIV in Kwara and Niger States. To align with the project's framework and ensure feasibility, purposive sampling was deemed the most appropriate and practical approach for this study. To further minimise potential bias, the study employed a multistage sampling technique for representativeness, and confidentiality was ensured during data collection to encourage candid responses.

**Instrument for data collection:** a semi-structured questionnaire was used to collect data, addressing each of the study objectives. It was written in English and translated into Hausa and Yoruba, the two predominant languages in Niger and Kwara State, respectively. The questionnaire had five sections, each addressing a variable of interest. Data was collected from October 2023 to February 2024, with quality ensured during collection, entry, and analysis. All 20 data collectors received comprehensive training to standardise interview techniques and ethical conduct. During the data collection period, six field supervisors, who were technical officers, conducted real-time, on-site checks of completed questionnaires. Any forms identified as incomplete or inconsistent were returned to the data collectors for immediate rectification, a proactive measure that minimised missing data and rendered subsequent imputation unnecessary. Finally, all questionnaires were systematically checked, numbered, and coded using a predefined guide before data entry to safeguard data integrity throughout the process.

**Validity and reliability:** several measures were taken to ensure instrument validity, including literature consultation, peer review, expert review, and pretesting in an ART clinic. Necessary corrections were made to ensure structure and content validity. The instrument, drafted in English, Yoruba, and Hausa languages, was pretested at different ART clinics not selected for the study. The reliability was determined using Cronbach's Alpha measurement, and the reliability coefficient was calculated on the pre-test questionnaire to ascertain the reliability of the instrument. For this study, the Cronbach Alpha was 0.9, which was considered to be reliable. Findings informed necessary corrections for relevance, context-friendliness, and appropriateness, ensuring a robust and reliable tool.

**Data management and analysis:** missing data were addressed at the point of data collection, where field supervisors checked questionnaires for completeness. Any incomplete questionnaires were returned to the data collectors for immediate correction with the participant. As a result, the dataset used for analysis was complete, and no imputation methods were required. Questionnaires were checked, numbered, and coded using a guide. Data were entered into STATA version 18 and analysed using descriptive statistics (frequencies, means) and inferential statistics (chi-square tests, logistic regression) to identify associations and predictors of vaccination and screening behaviour. Quantitative variables, including knowledge, practice, and risk perception scores, were summarised using descriptive statistics (means and standard deviations). For analytical purposes, these scores were categorised into groups (e.g., ‘poor´ (<33%), 'average' (33-66%), 'good' (>66%) knowledge) to facilitate the use of chi-square tests and to simplify the interpretation of results for public health intervention planning.

**Ethical considerations:** ethical approval was received from the Ethics Research Committee of the Kwara State Ministry of Health, which is licensed by the National Health Research Ethics Committee (NHREC) of Nigeria, and written permission was obtained from health facilities. Informed consents were collected from participants, ensuring confidentiality and anonymity. No personal identifiers were collected, and no physical harm or invasive procedures were involved. Respondents answered questions in a comfortable, private setting and could withdraw at any time without prejudice, aligning with international best practices for human subject research.

## Results

**Socio-demographic characteristics of the study participants:** the study included 991 WLHIV, comprising 539 (54.4%) from Kwara State and 452 (45.6%) from Niger State. The socio-demographic profile revealed notable differences between the two cohorts. In terms of religion, the majority in both States were either Christian or Muslim, with Kwara having a higher proportion of Muslims (62.5%) compared to Niger (54.6%), and Niger having a slightly higher proportion of Christians (44.7% vs. 37.3%). Educational attainment varied, with a higher proportion of participants in Kwara having tertiary or postgraduate education (44.6%) compared to Niger (32.3%). Conversely, a larger proportion of participants in Niger had only a primary-level education (33.6% vs. 20.2%).

Marital status also differed substantially, as the majority in Kwara were married (80.9%), whereas in Niger, 60.0% were married, and a higher proportion were not married/single (19.7%) or divorced/separated (12.8%). Residence distribution showed that over half of the Kwara participants lived in urban areas (52.5%), compared to 42.0% in Niger. Niger had a higher proportion of rural residents (34.1% vs. 27.8%). Key health-related behaviours showed significant disparities; a markedly higher proportion of participants in Niger (54.4%) reported ever being screened for cervical cancer compared to those in Kwara (25.0%). However, both States reported very low rates of HPV vaccination (Kwara: 4.1%; Niger: 3.1%) and a very low prevalence of past treatment for cervical pre-cancerous lesions (Kwara: 1.7%; Niger: 2.2%).

**Knowledge and awareness of cervical cancer among women of reproductive age living with HIV in Kwara and Niger States, Nigeria:** among 991 WLHIV, 539 (54.4%) from Kwara and 452 (45.6%) from Niger had heard of cervical cancer. Awareness of cervical cancer was most commonly obtained through family or friends in both States, reported by 56.4% of respondents in Kwara and 67.3% in Niger. Mass media was the second most frequent source (Kwara: 15.8%; Niger: 16.1%), followed by health workers (Kwara: 19.7%; Niger: 10.0%) and other sources (Kwara: 8.2%; Niger: 6.6%). The distribution of awareness sources differed significantly between the two States (X^2^ = 20.72, p < 0.001).

In Kwara, 38.4% knew it was a sexually transmitted infection, while only 23.7% in Niger were aware. Women in Kwara were more aware of multiple sexual partners (79.6%), early sexual activity (79.6%), and prolonged oral contraceptive use (77.0%) as risk factors. Those in Niger, however, recognised more factors, including poor diet (52.2%), family history (68.1%), early sexual activity (87.4%), prolonged oral contraceptive use (77.7%), and HPV (75.2%). For symptoms, 88.7% in Kwara and 89.4% in Niger knew intermenstrual bleeding was a symptom, and 87.6% in Kwara versus 95.8% in Niger knew about foul-smelling vaginal discharge as a key symptom. However, only 31.2% in Kwara and 25.4% in Niger were aware of different HPV strains. Furthermore, 93.1% in Kwara and 90.5% in Niger knew cervical cancer was preventable. Alarmingly, only 26.2% in Kwara and 40.7% in Niger knew cervical cancer screening should not be done annually. Finally, 77.9% in Kwara and 65.7% in Niger were aware of cervical cancer vaccination. Further details of knowledge of cervical cancer among WLHIV in North Central Nigeria are provided in Table 1.

**Table 1:** knowledge of cervical cancer among women of reproductive age living with HIV

Causes/risk factors of cervical cancer	Kwara (N = 539)		Niger (N = 452)	
	No	Yes	No	Yes
	n (%)	n (%)	n (%)	n (%)
Both males and females are at risk of cervical cancer	439 (81.4)	100 (18.6)*	384 (85.0)	68 (15.0)
Cervical cancer is a sexually transmitted infection	207 (38.4)*	332 (61.6)	107 (23.7)	345 (76.3)
Smoking	315 (58.4)	224 (41.6)*	192 (42.5)	260 (57.5)
Having multiple sexual partners	110 (20.4)	429 (79.6)*	52 (11.5)	400 (88.5)
Poor dietary intake	322 (59.7)	217 (40.3)*	216 (47.8)	236 (52.2)
Early sexual activity	110 (20.4)	429 (79.6)*	57 (12.6)	395 (87.4)
Family history of cervical cancer	252 (46.7)	287 (53.3)*	144 (31.9)	308 (68.1)
Prolonged use of oral contraceptives	124 (23.0)	415 (77.0)*	101 (22.3)	351 (77.7)
Human papillomavirus is a causative agent of cervical cancer	86 (16.0)	453 (84.0)*	112 (24.8)	340 (75.2)
Symptoms	-	-	-	-
Intermenstrual bleeding	61 (11.3)	478 (88.7)*	48 (10.6)	404 (89.4)
Foul-smelling vagina discharge	67 (12.4)	472 (87.6)*	19 (4.2)	433 (95.8)
Lengthy and heavier menstruation	67 (12.4)	472 (87.6)*	36 (8.0)	416 (92.0)
Pelvic pain	154 (28.6)	385 (71.4)*	70 (15.5)	382 (84.5)
Pain during intercourse	95 (17.6)	444 (82.4)*	53 (11.7)	399 (88.3)
Post coital bleeding	109 (20.2)	430 (79.8)*	72 (15.9)	380 (84.1)
Infertility	230 (42.7)	309 (57.3)*	42 (9.3)	410 (90.7)
Persistent diarrhoea	329 (61.0)	210 (39.0)*	222 (49.1)	230 (50.9)
Others	-	-	-	-
Aware of different strains of HPV	371 (68.8)	168 (31.2)*	337 (74.6)	115 (25.4)
Cervical cancer is preventable	37 (6.9)	502 (93.1)*	43 (9.5)	409 (90.5)
Cervical cancer is curable	209 (38.8)	330 (61.2)*	122 (27.0)	330 (73.0)
Heard about the pap smear test	299 (55.5)	240 (44.5)*	243 (53.8)	209 (46.2)
Cervical screening can help in the early detection of precancerous lesions	58 (10.76)	481 (89.2)*	33 (7.3)	419 (92.7)
Early detection of precancerous lesions and treatment can help in the prevention and treatment of cervical cancer	51 (9.5)	488 (90.5)*	21 (4.6)	431 (95.4)
Cervical cancer screening should be done annually	141 (26.2)*	398 (73.8)	184 (40.7)	268 (59.3)
A healthy woman should undergo a Pap smear test annually	192 (35.6)*	347 (64.4)	185 (40.9)	267 (59.1)
There is a vaccination for the prevention of cervical cancer	119 (22.1)	420 (77.9)*	155 (34.3)	297 (65.7)
Every woman, irrespective of age or sexual experience, should undergo cervical cancer screening	211 (39.1)*	328 (60.9)	121 (26.8)	331 (73.2)

* Indicates right response

The average knowledge score of participants from Kwara State regarding the causes, symptoms, and other matters related to cervical cancer was 17.92 ± 3.83 (95% CI = 17.60-18.25), while those from Niger State were 19.63 ± 3.09 (95% CI = 19.34-19.92). Based on the result, women living with HIV in Niger State significantly (t=7.61; p-value<0.001) had higher knowledge of the causes, symptoms, and other matters related to cervical cancer than their counterparts from Kwara State.

**Distribution of knowledge among participants in Kwara and Niger State:** concerning the distribution of knowledge among WLHIV from the two States, a very small proportion of participants from Kwara State (5.2%) had poor knowledge of cervical cancer. In addition, 367 (68.1%) had average knowledge, while the remaining 144 (26.7%) had good knowledge of cervical cancer. On the other hand, 0.4% of those from Niger State had poor knowledge, while 251 (55.5%) and 199 (44.0%) of participants in the State had average and good levels of knowledge regarding cervical cancer, respectively.

**Comparison of socio-demographic differences on associated factors:** the study found that educational attainment was significantly associated with knowledge levels, with the highest proportion of well-informed participants in Kwara having tertiary (40.3%) and secondary education (36.8%), similar to Niger, where tertiary (35.2%) and secondary education (33.2%) were linked to better knowledge. Location of residence was also significantly associated with knowledge in both States (p<0.001). However, the association between screening and knowledge differed: in Kwara, never being screened was linked to higher knowledge (75.7%, p=0.022), while in Niger, those screened had better knowledge (54.8%, p=0.301) (Table 2).

**Table 2:** comparison of socio-demographic differences on associated factors

	Kwara				Niger				
	Poor	Average	Good	p-value		Poor	Average	Good	p-value
**Religion**									0.052^a^
Christianity	8 (28.6)	144 (39.2)	49 (34.0)	0.527a		0	106 (42.2)	96 (48.2)	
Islam	20 (71.4)	222 (60.5)	95 (66.0)	-		2 (100.0)	145 (57.8)	100 (50.3)	
Others	0	1 (0.3)	0	-		0	0	3 (1.5)	
**Educational level**									0.003
Primary	19 (67.9)	67 (18.3)	23 (16.0)	<0.001		1 (50.0)	101 (40.2)	50 (25.1)	
Secondary	6 (21.4)	131 (35.7)	53 (36.8)	^a^		0	88 (35.1)	66 (33.2)	
Tertiary	3 (10.7)	148 (40.3)	59 (41.0)	-		1 (50.0)	56 (22.3)	70 (35.2)	
Postgraduate	0	21 (5.7)	9 (6.3)	-		0	6 (2.4)	13 (6.5)	
**Marital status**									0.392
Not married/single	1 (3.6)	35 (9.5)	13 (9.0)	0.114^a^		0	50 (19.9)	39 (19.6)	
Married	27 (96.4)	288 (78.5)	121 (84.0)	-		1 (50.0)	143 (57.0)	127 (63.8)	
Divorced/Separated	0	34 (9.3)	5 (3.5)	-		1 (50.0)	35 (13.9)	22 (11.1)	
Others	0	10 (2.7)	5 (3.5)	-		0	23 (9.2)	11 (5.5)	
**Place of residence**									<0.001
Rural	20 (71.4)	92 (25.1)	38 (26.4)	<0.001		1 (50.0)	111 (44.2)	42 (21.1)	
Sub urban/semi-urban	5 (17.9)	85 (23.2)	16 (11.1)	-		0	71 (28.3)	37 (18.6)	
Urban	3 (10.7)	190 (51.8)	90 (62.5)	-		1 (50.0)	69 (27.5)	120 (60.3)	
**Ever screened for cervical cancer**									0.301
No	27 (96.4)	268 (73.0)	109 (75.7)	0.022		2 (100.0)	114 (45.4)	90 (45.2)	
Yes	1 (3.6)	99 (27.0)	35 (24.3)	-		0	137 (54.6)	109 (54.8)	
**Ever being vaccinated against cervical cancer**									0.296
No	28 (100.0)	358 (97.5)	131 (91.0)	0.002^a^		2 (100.0)	246 (98.0)	190 (95.5)	
Yes	0	9 (2.5)	13 (9.0)	-		0	5 (2.0)	9 (4.5)	

**Difference in practice of cervical cancer screening and vaccination among participants:** measuring practice towards cervical cancer screening and vaccination of the participants revealed that the average practice score of those in Kwara State was 5.60 with a standard deviation of 1.50 (mean ± SD = 5.62 ± 1.50; 95% CI = 5.50 - 5.75). This average score was lower compared to that of participants in Niger State (mean±SD = 5.94±1.49; 95% CI = 5.81-6.08). The result also showed that the difference is statistically significant (t-value=3.34; p-value<0.001). Thus, this implies that participants in Niger State had significantly higher practice towards cervical cancer screening than those in Kwara State.

**Comparison of socio-demographic differences on factors that influence the practice of cervical cancer screening and vaccination among participants in Kwara and Niger State, Nigeria:** although practices towards cervical cancer screening differed in both states, findings from this study revealed some notable patterns. A significant association was found in Niger State between marital status and cervical cancer screening practice (p-value=0.046). Married women (64.8%) had better practices than single/married (17.4%) and divorced/separated (10.8%) women. In Kwara State, this pattern was similar but not significant (p-value=0.420). However, having been screened for cervical cancer was significantly associated with good practice in Kwara State (X^2^ = 107.48; p-value < 0.001) and Niger State (X^2^ = 52.11; p-value < 0.001), highlighting its importance in promoting cervical cancer screening and vaccination (Table 3).

**Table 3:** comparison of socio-demographic differences on factors that influence the practice of cervical cancer screening and vaccination among participants in Kwara and Niger State, Nigeria

	Kwara					Niger				
	Poor	Good	Total	χ^2^	p-value	Poor	Good	Total	χ^2^	p-value
**Religion**										
Christianity	108 (39.1)	93 (35.4)	201	1.83	0.373a	66 (40.0)	136 (47.4)	202	2.36	0.307
Islam	167 (60.5)	170 (64.6)	337	-	-	98 (59.4)	149 (51.9)	247	-	-
Others	1 (0.4)	0	1	-	-	1 (0.6)	2 (0.7)	3	-	-
**Educational Level**		-	-	-	-	-	-	-	-	-
Primary	44 (15.9)	65 (24.7)	109	6.62	0.085	54 (32.7)	98 (34.2)	152	0.34	0.952
Secondary	103 (37.3)	87 (33.1)	190	-	-	57 (34.5)	97 (33.8)	154	-	-
Tertiary	114 (41.3)	96 (36.5)	210	-	-	46 (27.9)	81 (28.2)	127	-	-
Postgraduate	15 (5.4)	15 (5.7)	30	-	-	8 (4.9)	11 (3.8)	19	-	-
**Marital status**	-	-	-	-	-	-	-	-	-	-
Not married/single	30 (10.9)	19 (7.2)	49	2.81	0.420	39 (23.6)	50 (17.4)	89	7.98	**0.046**
Married	220 (79.7)	216 (82.1)	436	-	-	85 (51.5)	186 (64.8)	271	-	-
Divorced/Separated	20 (7.3)	19 (7.2)	39	-	-	27 (16.4)	31 (10.8)	58	-	-
Others	6 (2.2)	9 (3.4)	15	-	-	14 (8.5)	20 (7.0)	34	-	-
**Place of Residence**	-	-	-	-					-	-
Rural	83 (30.1)	67 (25.5)	150	1.43	0.488	67 (40.6)	87 (30.3)	154	6.21	**0.045**
Sub urban/semi-urban	52 (18.8)	54 (20.5)	106	-	-	31 (18.8)	77 (26.8)	108	-	-
Urban	141 (51.1)	142 (54.0)	283	-	-	67 (40.6)	123 (42.9)	190	-	-
**Ever screened for cervical cancer**										
No	259 (93.8)	145 (55.1)	404	107.48	**<0.001**	112 (67.9)	94 (32.8)	206	52.11	**<0.001**
Yes	17 (6.2)	118 (44.9)	135	-	-	53 (32.1)	193 (67.2)	246		
**Ever being vaccinated against cervical cancer**					-	-	-	-	-	-
No	274 (99.3)	243 (92.4)	517	16.28	**<0.001**	163 (98.8)	275 (95.8)	438	3.07	0.079
Yes	2 (0.7)	20 (7.6)	22	-	-	2 (1.2)	12 (4.2)	14		
**Ever being treated for cervical cancer pre-cancerous lesion in the past**										
No	275 (99.6)	255 (97.0)	530	5.88	**0.015**	163 (98.8)	279 (97.2)	442	1.20	0.273
Yes	1 (0.4)	8 (3.0)	9	-	-	2 (1.2)	8 (2.8)	10		

WLHIV: Women Living with HIV

**Factors associated with cervical cancer screening among participants:** marital status was found to be significantly associated with screening in both Kwara (X^2^=20.82; p-value<0.001) and Niger State (X^2^=21.68; p-value<0.001). In Kwara State, only a few (2.0%) of the singles/not married had taken cervical cancer screening, compared to 26.4% and 28.2% of those who were married and divorced/separated, respectively. Elsewhere in Niger State, a lower proportion (33.7%) of the singles/not married, compared to 57.6% of those who were married and 69.0% of those who were divorced/separated, had taken the screening. The highest proportion of those who had taken cervical cancer screening in Kwara State was among urban dwellers (31.8%) compared to the lowest - rural dwellers (10.7%). However, in Niger State, those in semi-urban areas were those who predominantly (69.4%) took the screening, while the screening was least taken among urban settlers (49.0%). Both in Kwara (X^2^=23.70; p-value<0.001) and Niger (X^2^=13.01; p-value=0.001), dwelling location was a significant factor in cervical cancer screening. These results are presented in Table 4.

**Table 4:** socio-demographic factors associated with cervical cancer screening among participants

	Kwara				Niger			
	Ever had cervical cancer screening				Ever had cervical cancer screening			
	No	Yes	χ^2^	p-value	No	Yes	χ^2^	p-value
**Religion**	-	-	-	-	-	-	-	-
Christianity	146 (72.6)	55 (27.4)	1.21	0.518b	88 (43.6)	144 (56.4)	1.05	0.565
Islam	257 (76.3)	80 (23.7)	-	-	116 (47.0)	131 (53.0)	-	-
Others	1 (100.0)	0	-	-	2 (66.7)	1 (33.3)	-	-
**Educational level**	-	-	-	-	-	-	-	-
Primary	82 (75.2)	27 (24.8)	2.32	0.508	71 (46.7)	81 (53.3)	4.51	0.211
Secondary	137 (72.1)	53 (27.9)	-	-	62 (40.3)	92 (59.7)	-	-
Tertiary	164 (78.1)	46 (21.9)	-	-	66 (52.0)	61 (48.0)	-	-
Postgraduate	21 (70.0)	9 (30.0)	-	-	7 (36.8)	12 (63.2)	-	-
**Marital status**	-	-	-	-	-	-	-	-
Not married/single	48 (98.0)	1 (2.0)	20.82	**<0.001**	59 (66.3)	30 (33.7)	21.68	**<0.001**
Married	321 (73.6)	115 (26.4)	-	-	115 (42.4)	156 (57.6)	-	-
Divorced/Separated	28 (71.8)	11 (28.2)	-	-	18 (31.0)	40 (69.0)	-	-
Others	7 (46.7)	8 (53.3)	-	-	14 (41.2)	20 (58.8)	-	-
**Place of Residence**	-	-	-	-	-	-	-	-
Rural	134 (89.3)	16 (10.7)	23.70	**<0.001**	76 (49.3)	78 (50.7)	13.01	**0.001**
Sub urban/semi-urban	77 (72.6)	29 (27.4)	-	-	33 (30.6)	75 (69.4)	-	-
Urban	193 (68.2)	90 (31.8)	-	-	97 (51.0)	93 (49.0)	-	-
**Ever being vaccinated against cervical cancer**					-	-	-	-
No	400 (77.4)	117 (22.6)	39.37	**<0.001**	206 (47.0)	232 (53.0)	12.09	**0.001**
Yes	4 (18.2)	18 (81.8)	-	-	0	14 (100.0)		-
**Ever being treated for cervical cancer pre-cancerous lesion in the past**						-	-	-
No	404 (76.2)	126 (23.8)	27.39	**<0.001**	205 (46.4)	237 (53.6)	5.21	**0.022**
Yes	0	9 (100.0)	-	-	1 (10.0)	9 (90.0)	-	

WLHIV: Women Living with HIV

**Barriers to cervical cancer screening and vaccination among participants:** participants from Kwara and Niger States identified barriers to cervical cancer screening and vaccination. Fear of pain (59.7%, in both Kwara and Niger) and lack of awareness (57.9% in Kwara, 51.8% in Niger) were common barriers. Meanwhile, similar high proportions identified a lack of awareness about cervical cancer screening as a barrier in Kwara (63.3%) and Niger States (61.9%). Furthermore, many feared a positive screening result (58.4% in Kwara, 71.0% in Niger). Limited access to nearby screening facilities was a concern in both States (60.9% in Kwara, 53.8% in Niger). Few considered themselves too young for screening (19.9% in Kwara, 17.5% in Niger). Other barriers included fear of male screeners (67.5% in Niger, 48.8% in Kwara) and cost (51.6% in Kwara, 61.7% in Niger).

**Pattern of barriers among participants:** the study examined the pattern of barriers to cervical cancer screening and vaccination among participants from Kwara and Niger States. In Kwara, religion (p-value=0.548) and education (p-value=0.233) were not significantly associated with barrier levels. Though marital status was not significantly associated with the level of barrier among Kwara State participants (p-value=0.308), marital status was among those from Niger State (p-value=0.024). With married participants (61.8%) having higher barriers. Furthermore, the result showed that place of residence and screening uptake were significantly associated with the level of barrier among participants from both States. Place of residence and screening uptake were significantly associated with barrier levels in both States. Urban dwellers (50.7% in Kwara, 50.4% in Niger) had higher barriers. In addition, of all those in Kwara State who had a high-level barrier to cervical cancer screening and vaccination, the vast majority of them (82.3%) had never had cervical cancer screening. Similarly, in Niger State, an overwhelming 98.4% of those who had high-level barriers had never been screened for cervical cancer in the past (Table 5).

**Table 5:** pattern of barriers among participants

-	Kwara			Niger		
-	Low	High	p-value	Low	High	p-value
**Religion**	-	-	-	-	-	-
Christianity	93 (36.2)	108 (38.3)	0.548	93 (45.2)	109 (44.3)	0.903
Islam	164 (63.8)	173 (61.4)	-	112 (54.4)	135 (54.9)	-
Others	0	1 (0.3)	-	1 (0.5)	2 (0.8)	-
**Educational level**	-	-	-	-	-	-
Primary	61 (23.7)	48 (17.0)	0.233	80 (38.8)	72 (29.3)	0.056
Secondary	90 (35.0)	100 (35.5)	-	67 (32.5)	87 (35.4)	-
Tertiary	92 (35.8)	118 (41.8)	-	48 (23.3)	79 (32.1)	-
Postgraduate	14 (5.5)	16 (5.7)	-	11 (5.3)	8 (3.3)	-
**Marital status**	-	-	-	-	-	-
Not married/single	24 (9.3)	25 (8.9)	0.308	37 (18.0)	52 (21.1)	**0.024**
Married	212 (82.5)	224 (79.4)	-	119 (57.8)	152 (61.8)	-
Divorced/Separated	13 (5.1)	26 (9.2)	-	26 (12.6)	32 (13.0)	-
Others	8 (3.1)	7 (2.5)	-	24 (11.7)	10 (4.1)	-
**Place of residence**	-	-	-	-	-	-
Rural	49 (19.1)	101 (35.8)	**<0.001**	77 (37.4)	77 (31.3)	**<0.001**
Sub urban/semi-urban	68 (26.5)	38 (13.5)	-	63 (30.6)	45 (18.3)	-
Urban	140 (54.5)	143 (50.7)	-	66 (32.0)	124 (50.4)	-
**Ever screened for cervical cancer**		-	-	-	-	-
No	172 (66.9)	232 (82.3)	**<0.001**	72 (35.0)	134 (54.5)	**<0.001**
Yes	85 (33.1)	50 (17.7)	-	134 (65.0)	112 (45.5)	-
**Ever being vaccinated against cervical cancer**				-	-	-
No	248 (96.5)	269 (95.4)	0.516	196 (95.2)	242 (98.4)	**0.048**
Yes	9 (3.5)	13 (4.6)	-	10 (4.8)	4 (1.6)	-
**Ever been treated for cervical cancer pre-cancerous lesion in the past**					-	-
No	253 (98.4)	277 (98.2)	0.845	199(96.6)	243 (98.8)	0.117
Yes	4 (1.6)	5 (1.8)	-	7 (3.4)	3 (1.2)	-

WLHIV: Women Living with HIV

**Factors associated with screening uptake:** Table 6 presents the results of binary logistic regression regarding factors that are independently associated with screening uptake in Kwara and Niger States. As shown in the result, participants in Kwara State who were married are significantly more likely (OR=17.19; 95% CI = 2.34-126.01) than those who were not married/single to take part in cervical cancer screening. Also, those who were, although at one time married, but divorced or separated, are 18.85 times more likely (OR=2.31-153.91) than those who were single/not married to take the screening. Similarly, in Niger State, married participants (OR=2.66; 95% CI = 1.61-4.40) and divorced/separated participants (OR=4.37; 95% CI = 2.15-8.87) were more likely to take cervical cancer screening. The domicile of participants was also found to be significantly associated with screening uptake in both locations. Specifically, for participants in Kwara State, those in semi-urban areas had a 3.15 times higher likelihood (95% CI = 1.61-6.17) of screening uptake compared to those in rural areas. In addition, urban dwellers were 3.90 times more likely (95% CI = 2.19-6.94) than rural dwellers, to undergo cervical cancer screening. Meanwhile, those in Niger State, semi-urban dwellers had significantly higher odds (OR=2.21; 95% CI = 1.32-3.71) of screening uptake compared to their rural counterparts. Additionally, the result revealed that increased knowledge of cervical cancer among participants in Kwara State increased their chance of cervical cancer screening uptake. Based on the result, participants with average knowledge of cervical cancer (OR=9.97; 95% CI = 1.33-74.37), and those with good knowledge (OR = 8.66; 95% CI = 1.13-66.13) are more likely to take the screening. This was, although not the situation in Niger State, as their level of knowledge on cervical cancer had no significant association with their screening uptake. However, high barriers significantly reduced the chances of screening uptake in Kwara State (OR=0.43; 95% CI = 0.29-0.65) and in Niger State (OR = 0.44; 95% CI=0.30-0.65). Furthermore, among participants who resided in Kwara State, those with good practice towards cervical cancer had a 12.39 times higher likelihood (95% CI = 7.17-21.43) of screening uptake, while in Niger State, good practice also significantly increased the likelihood of screening uptake (OR=4.33; 95% CI = 2.88-6.53).

**Table 6:** factors associated with screening uptake

	Kwara				Niger			
-	OR	Lower	Upper	p-value	OR	Lower	Upper	p-value
**Religion**	-	-	-	-	-	-	-	-
Christianity	1.00	-	-	-	-	-	-	-
Islam	0.82	0.55	1.23	0.349	0.87	0.59	1.26	0.472
Others	-	-	-	-	0.38	0.03	4.32	0.440
**Educational level**	-	-	-	-	-	-	-	-
Primary	1.00				-	-	-	-
Secondary	1.17	0.68	2.01	0.557	1.30	0.82	2.04	0.255
Tertiary	0.85	0.49	1.46	0.564	0.81	0.50	1.29	0.382
Postgraduate	1.30	0.53	3.18	0.563	1.50	0.56	4.02	0.418
**Marital status**		-	-	-	-	-	-	-
Not married/single	1.00	-	-	-	-	-	-	-
Married	17.19	2.34	126.01	0.005	2.66	1.61	4.40	<0.001
Divorced/Separated	18.85	2.31	153.91	0.006	4.37	2.15	8.87	<0.001
Others	54.85	5.92	507.59	<0.001	2.80	1.24	6.32	0.013
**Place of residence**		-	-	-	-	-	-	-
Rural	1.00	-	-	-	-	-	-	-
Sub urban/semi-urban	3.15	1.61	6.17	0.001	2.21	1.32	3.71	0.003
Urban	3.90	2.19	6.94	<0.001	0.93	0.61	1.42	0.754
**Ever being vaccinated against cervical cancer**					-	-	-	-
No	1.00		-	-	-	-	-	-
Yes	15.38	5.10	46.34	<0.001	-	-	-	-
**Ever been treated for cervical cancer pre-cancerous lesion in the past**						-	-	-
No	1.00	-	-	-	-	-	-	-
Yes	-	-	-		7.78	0.97	61.96	0.053
**Knowledge of cervical cancer**			-	-	-	-	-	
Poor	1.00	-	-	-	-	-	-	-
Average	9.97	1.33	74.37	0.025	0.99	0.68	1.44	0.968
Good	8.66	1.13	66.13	0.037	1.00		-	-
**Barriers**	-	-	-	-	-	-	-	-
Low	1.00		-	-	-	-	-	-
High	0.43	0.29	0.65	<0.001	0.44	0.30	0.65	<0.001
**Practice**	-	-	-	-	-	-	-	-
Poor	1.00	-	-	-	-	-	-	-
Good	12.39	7.17	21.43	<0.001	4.33	2.88	6.53	<0.001

WLHIV: Women Living with HIV

**Risk perception of having cervical cancer among participants:** among WLHIV from Kwara State, most disagreed (37.3%) or strongly disagreed (52.3%) with the likelihood of being infected with cervical cancer in the future. A majority also agreed (34.9%) or strongly agreed (21.2%) that their risk of getting cervical cancer in the next few years is low, although 35.4% expressed fear about the disease. Additionally, 21.0% agreed and 5.7% strongly agreed that cervical cancer is incurable. Many participants (27.8% agreed, 18.7% strongly agreed) felt they were not at risk due to a lack of family history, despite 27.8% disagreeing and 14.3% strongly disagreeing with the perception that cervical cancer is not as common as projected, while 24.5% agreed and 6.1% strongly agreed with it. Most participants recognised the importance of protected sex, with 35.3% disagreeing and 24.9% strongly disagreeing that not using a condom with casual partners reduces their risk of cervical cancer (Table 7).

**Table 7:** risk perception of having cervical cancer among participants

	Kwara					Niger				
-	SA	A	U	D	SD	SA	A	U	D	SD
I feel it is likely that I will get cervical cancer in the future	5 (0.9)	12 (2.2)	39 (7.2)	201 (37.3)	282 (52.3)	10 (2.2)	17 (3.8)	60 (13.3)	125 (27.7)	240 (53.1)
My chance of getting cervical cancer in the next few years is low	114 (21.2)	188 (34.9)	52 (9.6)	104 (19.3)	81 (15.0)	70 (15.5)	86 (19.0)	80 (17.7)	112 (24.8)	104 (23.0)
The thought of cervical cancer scares me	35 (6.5)	191 (35.4)	104 (19.3)	101 (18.7)	108 (20.0)	65 (14.4)	141 (31.2)	144 (31.9)	60 (13.3)	42 (9.3)
Cervical cancer does not have a cure	31 (5.7)	113 (21.0)	98 (18.2)	168 (31.2)	129 (23.9)	28 (6.2)	63 (13.9)	100 (22.1)	180 (39.8)	81 (17.9)
If I developed cervical cancer, I would not live longer than 5 years	15 (2.8)	55 (10.2)	117 (21.7)	178 (33.0)	174 (32.3)	27 (6.0)	80 (17.7)	102 (22.6)	167 (37.0)	76 (16.8)
No one ever had cervical cancer in my family, hence, I’m not at risk	101 (18.7)	150 (27.8)	94 (17.4)	111 (20.6)	83 (15.4)	56 (12.4)	101 (22.4)	108 (23.9)	120 (26.5)	67 (14.8)
I feel having cervical cancer would threaten my relationship with my spouse	41 (7.6)	137 (25.4)	123 (22.8)	153 (28.4)	85 (15.8)	52 (11.5)	95 (21.0)	164 (36.3)	89 (19.7)	52 (11.5)
Multiple sexual partners cannot expose me to cervical cancer	43 (8.0)	100 (18.5)	108 (20.0)	172 (31.9)	116 (21.5)	24 (5.3)	55 (12.2)	92 (20.3)	146 (32.3)	135 (29.9)
Cervical cancer is not as common as projected	33 (6.1)	132 (24.5)	147 (27.3)	150 (27.8)	77 (14.3)	52 (11.5)	89 (19.7)	181 (40.0)	88 (19.5)	42 (9.3)
I am afraid of cervical cancer because we have family history	25 (4.6)	63 (11.7)	79 (14.7)	196 (36.4)	176 (32.7)	13 (2.9)	53 (11.7)	131 (28.9)	142 (31.4)	113 (25.0)
I have vaccinated myself against cervical cancer, I am not afraid of being infected	50 (9.3)	115 (21.3)	88 (16.3)	167 (31.0)	119 (22.1)	18 (4.0)	61 (13.5)	150 (33.2)	156 (34.5)	67 (14.8)
Even if I do not use condom when having sex with a casual person, I am not at risk	32 (5.9)	77 (14.3)	106 (19.7)	190 (35.3)	134 (24.9)	11 (2.4)	57 (12.6)	89 (19.7)	187 (41.4)	108 (23.9)

SA: Strongly Agree; A: Agree; U: Uncertain; D: Disagree; SD: Strongly Disagree

**Relationship between risk perception and cervical cancer screening and vaccination by women of reproductive age in the North Central State of Nigeria:** the study further examined whether a difference in risk perception exists among participants from the two different States. As shown in the result, the average risk perception of having cervical cancer was lower among participants from Kwara State (Mean±SD = 4.64±1.47; 95% CI = 4.51 - 4.76) compared to a higher average of 5.22±1.38 (95% CI = 5.09-5.34) among participants in Niger State.

Also, the result showed that WLHIV in Kwara State who had screening uptake had a lower average risk perception (mean±SD = 4.58±1.25; 95% CI = 4.37 - 4.79) compared to an average of (mean±SD = 4.65±1.54; 95% CI = 4.50 - 4.80) among those who did not have cervical cancer screening. However, the result also showed that the risk perception of those who had screen uptake was not significantly lower (p-value=0.619) than those who did not have screen uptake in the State. By comparison, the average risk perception of PLHIV in Niger State who had screen uptake (mean±SD = 5.16±1.47; 95% CI = 5.16±1.47) was, though lower, but not statistically different (p=0.326) from the average risk perception of their counterparts who did not have screen uptake (mean±SD = 5.29±1.27; 95% CI = 5.11-5.46).

## Discussion

The study reveals that most respondents were aware of cervical cancer, with a majority from Kwara State. This finding is consistent with Ogundipe *et al*. [13], where 66.55% of respondents had heard about cervical cancer among the university population in Ekiti State. However, it contrasts with a study in Lagos State by Olubodun *et al*. [14], which found that only 12.8% of participants were aware of cervical cancer and its screening. The study also examined sources of cervical cancer awareness among PLHIV, showing that family and friends are the primary sources of information in Kwara and Niger, similar to findings by Abudukadeer *et al*. [15] in China. This was followed by health workers and the mass media. While reliance on friends and family may lead to misinformation and insufficient information, leveraging mass media and healthcare professionals with adequate knowledge can significantly influence public beliefs and practices by raising awareness [16]. The results of this investigation corroborate earlier studies that have demonstrated that awareness of cervical cancer is not equivalent to a thorough knowledge of the disease [17]. While 76.2% of the women across both States in this current survey had heard about cervical cancer, only 34.6% across both States had adequate knowledge of the disease. Similar studies have shown that there is still a dearth of adequate knowledge about cervical cancer, with rates as low as 14.7% in Nigeria, 37% in Ghana, and 30% in China [15], among other countries. The inclusion of HIV-positive women in this study may have increased knowledge of cervical cancer since these women were more likely to have previously interacted with the healthcare system and to have been exposed to it.

The study found that most participants knew about HPV vaccination but had a limited understanding of specifics, which could impact vaccine uptake. Educational attainment was significantly linked to cervical cancer knowledge, with those having a tertiary education showing the highest proportion of good knowledge. This highlights the importance of higher education in enhancing awareness and understanding of cervical cancer. Ayeni *et al*. [18] reported similar findings, showing a positive correlation between higher educational attainment and better knowledge and attitudes towards cervical cancer screening. Additionally, urban residents across both States showed the highest levels of good knowledge, aligning with previous research that indicates urban residents have better access to health information and services, enhancing their understanding of diseases like cervical cancer [19].

The study also revealed a lower proportion of participants in Kwara State compared to Niger State who had been screened for cervical cancer or had ever had a Pap smear. This low screening rate is consistent with other studies in Nigeria, which have reported similarly low participation in cervical cancer screening programs. Ayeni *et al*. [18] found that despite good knowledge about cervical cancer, screening rates remain low due to factors such as fear, stigma, and lack of access to screening services, highlighting a significant gap between knowledge and practice. Moreover, only 9.3% in Kwara and 8.4% in Niger State had received the HPV vaccine, despite 72.4% awareness. This low uptake is alarming, mirroring trends in Nigeria and sub-Saharan Africa, where misconceptions, safety concerns, and limited availability hinder vaccination efforts [20]. Encouragingly, most participants intend to be screened for cervical cancer in the future, suggesting a positive attitude towards screening despite low current participation rates. This intention could be leveraged through targeted educational campaigns and improved access to screening services to increase actual screening rates [19]. Similarly, a large proportion of participants expressed a desire to be vaccinated against HPV, indicating a high level of acceptance and willingness to receive the vaccine if barriers such as cost, availability, and accessibility are addressed, as cited as key reasons for low uptake in this study.

Cervical cancer screening practices differed between Kwara and Niger States. Marital status influenced screening in Niger; a similar pattern was observed in Kwara State, though it was not statistically significant. Urban dwellers in Kwara were more likely to be screened (31.8%) compared to rural dwellers (10.7%). In Niger, semi-urban residents had the highest screening rates (69.4%), contrasting with urban residents' low rates (49.0%), highlighting disparities in access to health information and services. This finding aligns with previous research indicating that urban residents have better access to health information and services, enhancing their knowledge about diseases like cervical cancer [19].

Regarding barriers to uptake of cervical cancer screening and vaccination among participants in this study, a substantial proportion reported fear that screening is painful. This aligns with the study conducted by Mafiana *et al*. [21], who identified fear of pain as a common barrier to cervical cancer screening among Nigerian women. The fear of receiving a positive result is another major barrier in both States, often rooted in stigma and anxiety about the implications of a cancer diagnosis. This aligns with the study by Ubah *et al*. [22], where accessibility issues, such as the lack of nearby screening facilities and long waiting times, also pose significant barriers. These findings are consistent with Ezechi *et al*. [23], who noted that logistical challenges often deter women from participating in cervical cancer screening programs. Cultural factors, such as the need for spousal consent and concerns about male staff conducting the screening, were more significant barriers in Niger State compared to Kwara, reflecting broader socio-cultural dynamics influencing women's health behaviours. The cost of cervical cancer screening (62.0%) is a significant barrier, with financial constraints being a well-documented obstacle to accessing health services in many low- and middle-income countries [20]. In both Kwara and Niger States, high barriers to cervical cancer screening and vaccination led to low screening rates, with 98.4% of those facing barriers in Niger never being screened. This shows that barriers, if not effectively mitigated, could significantly deter cervical cancer screening and the possible uptake of the HPV vaccine for those eligible.

Finally, in Kwara State, increased knowledge of cervical cancer boosted screening likelihood, with those having average or good knowledge more likely to participate. However, in Niger State, knowledge level didn't impact screening uptake. High barriers reduced screening chances in both States. Good practices towards cervical cancer significantly increased screening uptake in both States, with a 12.39 times higher likelihood in Kwara.

**Limitations:** the findings are based on a facility-based sample of women already engaged in HIV care, which may not be generalizable to all women living with HIV in the community. Furthermore, the cross-sectional design captures data at a single point in time, preventing causal inferences.

## Conclusion

The study highlights significant gaps in cervical cancer prevention despite high educational attainment. Many participants knew of cervical cancer, but comprehensive knowledge was low, and screening/vaccination uptake was alarmingly deficient. Key barriers included fear, lack of awareness, logistical challenges, and cultural factors. Public health professionals must address these barriers to increase uptake of cervical cancer prevention and treatment services. The study emphasises the importance of education in enhancing awareness and promoting positive health practices. Urban residency and higher education were linked to better knowledge and practices, highlighting demographic disparities. To address this, comprehensive educational campaigns should be implemented, targeting both urban and rural areas. Increasing access to services through mobile clinics and community outreaches can also help. Additionally, male involvement in cervical cancer prevention initiatives, such as supporting spousal screening and vaccination, should be encouraged. Healthcare providers should be trained to educate and encourage women to utilise screening and vaccination services.

### What is known about this topic

- Cervical cancer is the fourth most common cancer in women, primarily caused by persistent HPV infection;
- Women of reproductive age are at risk of cervical cancer.

### What this study adds

- Awareness is good, but actual knowledge about cervical cancer, especially screening and prevention, is very low among PLHIV;
- Despite the increased vulnerability of women living with HIV, screening and vaccination are very poor;
- A huge number of women, 96.3% had never been vaccinated.
